# Narrative Methods and Sociocultural Linguistic Approaches in Facilitating In-depth Understanding of HIV Disclosure in a Cohort of Women and Men in Cape Town, South Africa

**DOI:** 10.3389/fpubh.2016.00095

**Published:** 2016-05-17

**Authors:** Diane Cooper, Joanne E. Mantell, Ntobeko Nywagi, Nomazizi Cishe, Katherine Austin-Evelyn

**Affiliations:** ^1^School of Public Health, University of Western Cape, Cape Town, South Africa; ^2^Division of Gender, Sexuality and Health, HIV Center for Clinical and Behavioral Studies, New York State Psychiatric Institute and Department of Psychiatry, Columbia University Medical Center, New York, NY, USA; ^3^Women’s Health Research Unit, School of Public Health and Family Medicine, University of Cape Town, Cape Town, South Africa; ^4^International Women’s Health Coalition, New York, NY, USA

**Keywords:** HIV disclosure, researcher–participant interactions, narrative method, qualitative cohort, language and culture, South Africa

## Abstract

The South African National Department of Health has rapidly extended free public-sector antiretroviral treatment for people living with HIV from 2007. Approximately 6 million people are living with HIV in South Africa, with 3.1 million currently on treatment. HIV disclosure stigma has been reduced in high prevalence, generalized epidemic settings, but some remains, including in research interviews. This paper documents the unexpected reactions of people living with HIV to interviewers. It highlights shifts over time from discussing daily events with researchers to later expressing distress and then relief at having an uninvolved, sympathetic person with whom to discuss HIV disclosure. While there are commonalities, women and men had gendered responses to interviewers. These are apparent in men’s uncharacteristic emotional responses and women’s shyness in revealing gendered aspects of HIV acquisition. Both women and men expressed stress at not being allowed or able to fulfill dominant expected masculine or feminine roles. The findings underline the role of research interviewers in study participants confiding and fully expressing their feelings. This greater confidence occurred in follow-up interviews with researchers in busy health facilities, where time of health-care providers is limited. It underlines the methodological value of narrative inquiries with research cohorts. These allowed richer data than cross-sectional interviews. They shaped the questions asked and the process of interview. They revealed participants’ increasing level of agency in expressing feelings that they find important. This research contributes to highlighting pivotal, relational aspects in research between empathetic, experienced researchers and study participants and how participant–researcher relationships progress over time. It highlights ethical dilemmas in roles of researchers as opposed to counselors, raising questions of possible blurring of lines between research and service roles. This requires further research exploration. It additionally underscores the importance of “care for the carer.” Furthermore, it emphasizes that cultural sensitivity to language involves more than merely speaking the words in a language. Culture, humor, dialects, conceptual issues, wordplay, common sense, and respectful attitudes to other languages, resonates.

## Introduction

“At its heart, public health is a conversation society has …” [(1), p. 3].

The South African National Department of Health has rapidly extended free public-sector antiretroviral treatment for people living with HIV (PLWH) from 2007. The country has the largest ART treatment program in the world (2). Approximately 6 million people are living with HIV in South Africa, with 3.1 million currently on treatment. Those PLWH currently qualifying for free life-long treatment include PLWH whose CD4^+^ count is <500, pregnant women living with HIV (WLWH), those with a repeat episode of TB, children, or having AIDS-related symptoms (3). If South Africa transitions to free ART regardless of CD4^+^ status, as WHO now recommends (4), this number will double. As HIV has become more common in generalized high prevalence epidemics such as in South Africa, stigma has been reduced.

The pattern of HIV disclosure among adults is likely to be selective over time. Disclosure to health-care providers, intimate partners, and chosen family members and friends is most common (5). Less common is broader openness of HIV status in residential communities, except where PLWH have a high level of institutional support, HIV activism, and advocacy (6). Disclosure is conditional on a number of factors, including individuals’ perceptions of their own socioeconomic status in the community. Frequently, those with lower and higher socioeconomic status are more reluctant to disclose. Disclosure in the workplace is still uncommon, despite legislation in South Africa that prohibits discrimination (7). The dynamics of HIV status disclosure and issues of stigma is a much-studied topic. However, as the terrain of availability and access to treatment changes, it warrants continued research.

This article seeks to continue the conversation on disclosure by acknowledging how this process plays out in dynamic interactions between research participants and researchers. This is specific to research methods used and is situated within broader sociocultural and language contexts. The focus of this article is on the experiences and practices of qualitative researchers in discussing HIV disclosure with a cohort of women and men living with HIV. This took place during a period when access to ART was being increased. It documents participants’ unexpected reactions to interviewers captured in sometimes, gendered “distress” and “well-being” narratives and their support needs. Additionally, it underscores the importance of relations between participants and researchers (8) in a qualitative, narrative cohort study and of sociocultural linguistic understandings.

## Materials and Methods

### Study Design and Procedures

The study used a qualitative narrative methodology, with data collected through in-depth interviews (IDIs) with a cohort of women and men living with HIV. This was prior to implementation of a multilevel structural intervention study integrating sexual and reproductive health issues into HIV care. These interviews assisted in analyzing participants’ subjective meanings and reactions to disclosure. Qualitative methods are inductive and search for meaning rather than measuring trends, proportions, or patterns of association. They place emphasis on human discourses (9).

Three interviews were conducted between 2007 and 2012 with approximately 9-month intervals between interviews. Participants newly diagnosed with HIV were recruited for the first interview from four HIV care clinics. These clinics serve clients with a demographic profile likely to be seen in other HIV care clinics in Cape Town’s public health sector.

Qualitative interviewers were experienced, same-sex, and English fluent, first-language *isiXhosa*-speaking researchers. Semi-structured interviews were conducted in participants’ preferred language. Initial interviews were conducted with 30 women (age range 19–61 years) and 27 men (age range 20–53 years) living with HIV. Second interviews were conducted with 23 women and 20 men, and third interviews with 20 women and 19 men. Baseline interviews without follow-up were excluded from this analysis. Loss to follow-up was primarily due to being too ill for interview, deaths, or moving out of the study area. A small number refused a follow-up interview or were untraceable. The same female and male interviewers interviewed participants at baseline and follow-up interviews.

Interviewers were encouraged to remain emotionally neutral and suggest interventions or referrals when the interview was complete.

Interviews were approximately 1.5 h in length, audio-recorded, and subsequently transcribed by the same interviewers. In the baseline interview, we asked the following questions: “After learning you were HIV^+^, who did you feel you could talk to about your HIV status, if anyone, and how did you go about talking with them?” “How did they react to your having HIV?” In the second interview, we asked: “Since you were last interviewed, have you changed how you feel about telling (more) people about your HIV?”

At the final interview, we asked: “Since our last interview, has there been anyone new that you have talked to about your having HIV?” Most interviews took place in a public-sector health service environment, attended by 84% of the population (10). Interviewers had regular group debriefings with the Principal Investigator to discuss their own feelings, how they had dealt with them, and strategies for addressing issues that emerged.

### Ethics

The nature of the research project was explained to potential participants in writing and verbally in English and *isiXhosa*, and written informed consent obtained. Potential participants could refuse participation or withdraw at any stage without any repercussions. Participants’ names and identities were protected. The Health Sciences Faculty Human Research Ethics Committee at the University of Cape Town and the Institutional Review Board at the New York State Psychiatric Institute – Columbia University Department of Psychiatry approved the study.

### Analysis

In this article, we use participant IDIs, but also rely on interviewers’ written field notes, experiences, practices, and minutes from meetings. All data were incorporated, managed, and coded using the Nvivo software package.

Using a coding list, thematic and narrative analysis was used to highlight how women and men reacted to HIV disclosure to researchers (11). The participants’ narratives were examined in a sequence of events: (i) initial reactions to talking about disclosure experiences with interviewers, (ii) later confidences to interviewers, and (iii) disclosure-related counseling needs that arose from interviewer interactions. Reliability of interpretation of issues emerging was checked with the two researchers who conducted the interviews and another researcher. In addition, interviewer’s written field notes and meeting minutes were compared with transcripts of interviews.

## Results

Table 1 provides a demographic profile of participants.

**Table 1:** **Participants’ characteristics**.

	Female (aged 1–61 years)	Male (aged 20–53 years)
# participants at baseline interview	30	27
# participants at interview 2	23	20
# participants at interview 3	20	19
Relationship status (currently has main sexual partner)	76% (35% in casual relationships)	84% (58% in casual relationships)
Mean age	33 years	37 years
Mean education	10.4 years	9 years
Time since HIV diagnosis	2 weeks–6 months	2 weeks–6 months
Dates of interviews	October 28, 2007–February 28, 2012	November 1, 2007–July 30, 2012

Participants’ discussion about HIV disclosure with interviewers was a dynamic process. This changed from initially one of distance to one of trust invested in the interviewers over time. The quotations reflect key issues that emerged. All names used are pseudonyms.

### First Interview – “Distance”

At first interview, many participants had not disclosed their HIV status outside of the health-care environment. The manner of narrating disclosure to interviewers tended to be dispassionate and distanced. They typically dealt with the daily realities of taking medication and attending services. For example, Sizwe, a 50-year-old man said

Now I just think about taking my medication to keep me well. I am glad I have told my wife about my condition as she helps me to remember.

Nompendulo aged 18 reflected:

I have to make up a reason why I go to the clinic for my ‘Beco’ [Vitamin B complex], as my family doesn’t know, but I just keeping on concentrating on keeping myself well.

### Second Interview – “Part Distance” Covered and Unexpected Issues Arise – Narratives of Distress

By the second interview, participants engaged in an emotional process of investing confidence in the interviewers. Narratives of heightened dismay, feelings of exclusion from family decision-making, stigma, and other issues emerged spontaneously.

Men, in particular, became emotional during the second interview. Tearfulness about their feelings of loss of status as men in family decision making was common. Sizwe, a 50-year-old man, wept as he said

Usually in our culture, older men like myself are included in all decisions about the larger family. Since I told them I have HIV, I am alone. I am not invited.

He expressed discomfort as a man, in crying. Nevertheless, he was comforted when the interviewer listened quietly and patiently, allowing him to express himself fully. Thabo, 35 years old, shared with the interviewer feelings of great sadness in being incapable of providing economically for his family due to illness. He was unable to share these feelings with his family.

Jonga, 28 years old, became very emotional during the second interview. He said that he wanted to go home to tend to family cattle in which men are generally involved. However, he was too sick and feared disclosing his HIV status telephonically to his family. He received comfort from the researcher. After the interview, the interviewer counseled him on preparing to disclose his status to his family.

Nompendulo, aged 18, feared adverse and judgmental reactions from her family if she disclosed her HIV status. Initially, she was reluctant to discuss this in any depth with the researcher.

A shared theme among women and men was great distress in sharing their experiences of disclosure to researchers, beyond their comfort zone. For example, 40-year-old Nomsa painfully related that her husband had divulged her HIV status to the church minister and congregation in a rural area where she resided. When she relocated to Cape Town to avoid the stigma she experienced as a result of her husband’s unsolicited disclosure, she experienced further trauma. Her husband had disclosed her status to the new urban minister who, in turn, disclosed this to the new pastoral community. During the interview, the interviewer sympathized. After the interview, she offered to talk with the church minister. Men tended to experience greater discomfort than women at first interview in sharing their feelings with researchers, but changed by the second and third interviews.

### Third Interview: “Going the Full Distance” – Narratives of Relief and Gender

By the third interview, participants expressed relief in having someone who was not a family member or friend with whom to talk. Thando, a 32-year-old, gained comfort in speaking to the interviewer:

I feel there is a change because I feel right now – I can speak to you about it … but at the beginning, I was isolated and it was painful.

By the third interview, Jonga, who was mentioned earlier, was on ART. He said he felt joyful and relief at the advice the interviewer had given him. He had disclosed to his family and was ready to return to his family’s rural residence.

As mentioned, Nompendulo was reluctant to discuss her reservation about disclosure with the interviewer initially. She thawed at the third interview, admitting that she thought she may be judged by the researcher as a female, having been sexually active at 15 years. At the third interview, she responded well to the researcher’s suggestion that she “test the waters” by first finding out how different family members would react to someone in the family living with HIV. Watching a TV program with her family in which HIV arose was agreed as a good way to initiate this.

Xoliswa, aged 23, who lived with her boyfriend said:

When we talked earlier I thought about my being HIV positive because I need not to keep this to my heart, I need to tell somebody. It was easy with you, I lost my stress to talk, because you saw me time and again.

Phindiwe, aged 27, currently with no boyfriend said she was reluctant to disclose her HIV status broadly:

Because people usually think, especially as a woman, that its because you misbehaved whereas sometimes its not mischief because even yourself you do not know where did you get virus, I thought a first even you might think this but I was wrong and it was good to talk with you. You listened without worrying if it took time.

### Language and Culture: of Bulls, Cows, Calves and Pigs

Participants commonly used metaphors in relating feelings. Monde, a 47-year-old man, used the term “bull,” “cows,” and a disabled “pig” to denote his dismay in exclusion from family decision making:

I used to be a bull, but now I am a cow among the bulls – they don’t take me with them anymore. … those who are left [are like me] … a castrated pig in the house [valuable livestock, but ‘impotent’ in important matters].

Thandi, 35 years old, spoke about her discomfort despite being unwell, in not being able to fulfill gendered expectations of being able to work, care for children, and complete domestic chores. She used the phrase “letting the calf go to its mother.” This term of speech indicated that it appeared she was fulfilling her roles while not doing so to avoid disclosure. However, she was willing to discuss her misgivings with respect to fulfilling gender roles and disclosure with the researcher.

## Discussion

At all three interview periods, participants’ discussion of disclosure to researchers differed. Management of the disclosure process and style changed. This was reflected in the differences in participants’ willingness to reveal thoughts and feelings.

### Reflections: Disclosure Journey and Researcher Practices

As mentioned, men and women showed specific vulnerabilities in narrating their experiences of HIV disclosure. Stigma and distress were therefore sometimes gendered. This mirrored differences documented elsewhere with respect to HIV (12, 13). Men frequently feel they should not show emotions (12–14). However, male participants’ uncharacteristically emotional narratives during later interviews were contrary to perceptions of normative, dominant male behavior. In contrast, men continued to identify with dominant masculine roles associated with being heads of household (15–17). Their role as decision makers in patrilineal extended families was perceived as critically important to their social and personal status. In their second interviews, men found a safe space to be very emotional about such issues as being stripped of their roles in long-term decision making in families and clans. This occurred despite shifts socially to HIV’s normalization and perceived manageability. Their anxious, insecure, and sad behavior contrasted with their previously dominant male persona in an ongoing interview environment (16).

Mfecane argues that “if research is a social practice, then making friends in the field is a productive, sometimes essential strategy, the more comfortable they feel with us as researchers, the more insightful our research findings are likely to be” [(17) p125]. This was evident in our research. However, unexpected counseling tasks were frequently inadvertently “delegated” to the interviewers. The context-specific ethical dilemmas facing researchers are underscored in this study. While the researchers in our study may have been tempted to leap in and offer support as opposed to maintaining emotional neutrality during an interview, they had been trained to avoid this and leave any necessary intervention until after the interview. We had prepared a list of referral persons and organizations for researchers to offer participants, if they wished. This highlights issues raised in public health and social science research about the “situational complexities of ethical decision making as they arise somewhat unpredictably in the field and the very personal ways in which researchers had to deal with them; in the heat of the moment and then as this cools with introspection” [(18), p. 6]. The ethical issues of maintaining a distance between researchers and participants in the field require further research exploration.

### Reflections: Cultural–Linguistic Issues

Important cultural–linguistic issues influencing the narratives emerged. Thematic and narrative analysis pays attention to what participants say, the process of story telling, the impact on what emerges, and the manner of retelling. Discourse analysis focuses on language, and how this reflects cultural and social linguistics. Phrases participants used reflect sociocultural elements to capture identity, experiences, as well as categories and labels (19, 20). Without a discourse analysis, we “skimmed the surface” in how language impacts. Qualitative cohort data lend itself to future discourse analysis. Hunter (21) and Dowling (22) highlight cultural meanings and “lost in translation” misunderstandings that occur in translating *isiXhosa* to English and *vice versa*. This underscores the key role of English fluent first-language speakers in continuity in interviewing, translation, and analysis. Similar to English in which we have the expression “take the bull by the horns,” which is not literal but rather denotes confronting issues head on, in *isiXhosa*, there are different expressions about bulls with completely different meanings.

Both Dowling (22) (with respect to *isiXhosa*) and Epprecht (23) emphasize that translations or their understanding may meander far from their original meanings (22, 23). Dowling (22) singles out medical terminology, in giving an example of a medical questionnaire that needed the participant to choose the answer that “fits” where the meaning in i*siXhosa* translated to “epileptic fits.” The use of metaphors by our participants about “pigs” and “cows,” if taken literally, underscore these points. Culturally, specific references to bulls (males) being stronger and more competent than cows (females) predominated in these narratives. However, cows are also a critically important resource among *amaXhosa*, a source of wealth and bride-wealth (*lobola*). The latter can take on different meanings in “living” custom. Some men may interpret this as meaning “ownership” of a wife, while others may see this as needing to “treasure” a wife. Pigs in *isiXhosa*-speaking communities do *not* denote dirty or taboo animals. They are an appreciated economic resource. However, in this context, the addition of the term “castrated” gives them a different meaning. Our male research interviewer commented that if an analysis that misunderstood the cultural–linguistic underpinnings, the respondent would not recognize the interpretation of what he had originally said in the interview. Furthermore, the phrase of “letting the calf go to its mother” would be misunderstood unless the meaning from the *isiXhosa* translation was clear.

First-language *isiXhosa*-speaking experienced interviewers were able to understand the nuances in language and tune in to sociocultural linguistics. They uncovered hidden and spontaneous meanings. Similarly, in microbicide research, sexual violence being a reason for microbicides spontaneously emerged (24). In HIV and abortion research, the latter was perceived as much more stigmatized than HIV (25). Experienced and trained interviewers, aware of their researcher positions and socially and culturally sensitive, elicited often otherwise hidden reactions. They were thus able to console and later counsel appropriately. This assists us in cautioning against “parachute research” in which researchers unfamiliar with Xhosa culture may sometimes conduct research or analysis without understanding language or other issues within a specific context [(26), p. 101].

### Limitations

There are several limitations. Reports are necessarily retrospective and subject to recall problems. This may affect the reliability of the narratives of disclosure to others, but not their emotions in interviewer discussions, which were immediately noted. We minimized English translation bias by having bilingual, *isiXhosa* first-language speakers interviewing, transcribing, translating, and participating in analysis. The aim of qualitative research is to produce rich insights and depth rather than breadth in its findings. “‘Transferability’ in this context means developing a theory that may determine or constitute broader social phenomena” [(27), p. 247]. Reflexivity, critical in qualitative research, involved steps to minimize and acknowledge researchers’ own views that may intrude in data collection and analysis.

## Conclusion

HIV disclosure, when and to whom, forms an integral part of the lives of people living with HIV. Disclosure is not always a good thing and sometimes may not make logical sense rather than being a reaction to stigma. People frequently weigh up situations and make strategic decisions in this regard that have favorable outcomes for them. People take a meandering rather than a linear path in disclosure. Importantly, health-care providers, researchers, and many others they meet influence them along the way. Qualitative research interviewers are often able to spend more time with health service clients they interview than health-care providers in busy public-sector health facilities, particularly when they conduct follow-up interviews. Participants are able, as a result, to confide in them about disclosure and express their emotions fully. They can play an important role in participants’ well being and moving forward to further disclosure, where this is the correct decision for them.

The findings underline the value of narrative inquiries with research cohorts in allowing richer data than in cross-sectional interviews. In addition, participants’ increasing level of agency in being able to discuss what they feel is important and express their feelings to researchers is highlighted (28). Individuals’ reactions on disclosure to researchers shaped the topic at hand (11) and raised questions that researchers might not have thought to ask and perhaps uncovered what participants did not initially intend to disclose (29). Issues of moving from daily concerns related to disclosure to expressing distress and later to relief are highlighted. Although there were commonalities between men and women, a pattern of gendered differences in responses is clear.

The research highlights pivotal, relational aspects in research between empathetic and experienced researchers, whose first language is the same as participants, and the manner in which participant–researcher relationships progress over time. Male participants disclosed distressing, emotional disclosure experiences to interviewers, sometimes contrary to gendered expectations. A series of interviews with the same participants revealed modified, and shifting narratives remind us once again: “At its heart public health is a conversation society has …” [(1), p. 3]. Furthermore, it underscores the importance of research interviewers who may need to counsel or debrief study participants at the end of an interview, having the necessarily skills, empathy, and understanding to do so. The process also continues to raises questions where lines may blur between research and service roles, and the experience needed in whether or not to counsel after the interview or refer participants to expert persons or organizations. Hekman’s argument on agency captures this succinctly: “The elements of the mangle are mangled; they are mixed up with each other into a combination in which the various elements lose their clear boundaries” [(30), p. 24].

In addition, the importance of language beyond merely speaking the words in a language is highlighted. In research, Dowling’s (22) call to ensure that we consider culture, humor, dialects, conceptual issues, word play, common sense, and respectful attitudes to other languages, resonates.

## Author Contributions

DC was South African Principal Investigator, did the primary analysis for this paper, and led this paper. JM was the Principal Investigator in the USA and contributed to the writing of this paper. NN was a key researcher involved in male interviews for study, participated in the interpretation of analysis, and contributed to the writing of the paper. NC was a key researcher involved in female interviews for study, participated in interpretation of the analysis, and contributed to the writing of the paper. KA-E was a Master in Public Health student at the University of Cape Town. She participated in the literature review, the data analysis, and writing up as part of this study under the auspices of the Women’s Health Research Unit at UCT. All authors reviewed and commented on all drafts, and ratified the final paper submitted.

## Conflict of Interest Statement

The authors declare that the research was conducted in the absence of any commercial or financial relationships that could be construed as a potential conflict of interest.
